# HMGB1‐induced angiogenesis in perforated disc cells of human temporomandibular joint

**DOI:** 10.1111/jcmm.13410

**Published:** 2017-10-30

**Authors:** Yaping Feng, Jin Ke, Pinyin Cao, Mohong Deng, Jian Li, Hengxing Cai, Qinggong Meng, Yingjie Li, Xing Long

**Affiliations:** ^1^ State Key Laboratory Breeding Base of Basic Science of Stomatology (Hubei‐MOST) Key Laboratory of Oral Biomedicine Ministry of Education (KLOBM) School and Hospital of Stomatology Wuhan University Wuhan Hubei China; ^2^ Department of Oral and Maxillofacial Surgery School and Hospital of Stomatology Wuhan University Wuhan Hubei China

**Keywords:** HMGB1, temporomandibular joint, perforated disc cells, VEGF, HIF‐1α, Erk, P38, JNK

## Abstract

High mobility group 1 protein (HMGB1), a highly conserved nuclear DNA‐binding protein and inflammatory mediator, has been recently found to be involved in angiogenesis. Our previous study has demonstrated the elevation of HMGB1 in the tissue of perforated disc of temporomandibular joint (TMJ). Here, we investigated a novel mediator of HMGB1 in regulating hypoxia‐inducible factor‐1α (HIF‐1α) and vascular endothelial growth factor (VEGF) to mediate angiogenesis in perforated disc cells of TMJ. HMGB1 increased the expression of HIF‐1α and VEGF in a dose‐ and time‐dependent manner in these cells. Moreover, immunofluorescence assay exhibits that the HIF‐1α were activated by HMGB1. In addition, HMGB1 activated extracellular signal‐related kinase 1/2 (Erk1/2), Jun N‐terminal kinase (JNK), but not P38 in these cells. Furthermore, both U0126 (ErK inhibitor) and SP600125 (JNK inhibitor) significantly suppressed the enhanced production of HIF‐1α and VEGF induced by HMGB1. Tube formation of human umbilical vein endothelial cells (HUVECs) was significantly increased by exposure to conditioned medium derived from HMGB1‐stimulated perforated disc cells, while attenuated with pre‐treatment of inhibitors for VEGF, HIF‐1α, Erk and JNK, individually. Therefore, abundance of HMGB1 mediates activation of HIF‐1α in disc cells *via* Erk and JNK pathway and then, initiates VEGF secretion, thereby leading to disc angiogenesis and accelerating degenerative change of the perforated disc.

## Introduction

Disc perforation, widely accepted as the most seriously pathologic subtype in osteoarthritis of temporomandibular joint (TMJOA), is one of the main causes of joint pain, limited mouth opening and cartilage degeneration 1. The underlying mechanism of disc perforation has been attributed to diverse factors including aberrant occlusal forces, sexual difference and accelerated chondrocyte‐like apoptosis 2, 3. Although multiple researchers attempted to address this issue, the specific mechanism of disc perforation remained unclear.

Anatomically, the body of the TMJ disc is avascular in adult humans 4. In contrast, a high density of blood vessels occurs in perforated discs vicinity either during discectomy or under arthroscopy 5, 6, indicating vascularization may exert a key role in disc perforation. Indeed, current attention has switched to the relationship between angiogenesis and disc degeneration. Kiga *et al*. reported that neovascularization, accompanied with the accumulation of VEGF, correlates with the severity of deformed discs. They suggested that VEGF initiates proliferation and chemotaxis of endothelial cells, augments vascular permeability and induces the secretion of matrix metalloproteases (MMPs), which attribute to the degradation of extracellular matrix of discs 7. Agrawal *et al*. indicated that enhanced vascular invasion boosted by VEGF and HIF‐1α contributes to degenerative changes in the intervertebral disc 8.

High mobility group box 1 (HMGB1), a highly conserved and widely expressed protein, is distributed in the kidney, heart, lung, brain, liver and has important role in various of pathological and physiological processes 9, 10. In the nucleus, HMGB1 exerts multiple functions, including the regulation of genome replication, recombination, mRNA transcription and DNA repair 11. HMGB1 can also be actively secreted by immunological cells or passively released by necrotic cells. Once released in the extracellular space, HMGB1 can induce a series of reactions such as inflammation and angiogenesis 12, 13. It has been proven that extracellular HMGB1 promotes angiogenesis in various somatic tissues through bonding to various receptors 14. Youn *et al*. have pointed out that HMGB1 can exert angiogenic effects through induction of the activity of HIF‐1α in synovial fibroblasts from rheumatoid arthritis 15. Additionally, HMGB1 was reported to promote angiogenesis in collagen antibody‐induced arthritis *via* a VEGF‐dependent mechanism 16. Our previous study has demonstrated the elevation of HMGB1 in the tissue of perforated disc of TMJ 17. Therefore, it is reasonable to assume that HMGB1 may participate in the pathologic process of perforated discs through angiogenic induction *via* HIF‐1α and VEGF. Nevertheless, the specific mechanism that HMGB1 evokes angiogenesis in perforated disc of TMJ has been unknown yet.

This study therefore was designed to investigate whether HMGB1 regulated HIF‐1α‐linked VEGF expression in perforated disc cells derived from human TMJ. Additionally, we further explore the potential signal pathways by which HMGB1 up‐regulates HIF‐1α in these cells.

## Materials and methods

### Reagents and antibodies

Recombinant human HMGB1 purchased from Sigma‐Aldrich (St. Louis, MO, USA) and specific chemical inhibitors including BAY87‐2243, U0126, SB203580 and SP600125 were purchased from Selleck (Houston, TX, USA). Antibodies for phospho‐Erk (Thr202/Tyr204,p‐ERK), total Erk, phospho‐p38 MAP kinase (Thr180/Tyr182,p‐p38), total P38 MAP kinase, phospho‐JNK (Thr183/Tyr185, p‐JNK) and total JNK were purchased from Cell Signaling Technology (Danvers, MA, USA). Antibodies for HMGB1 and HIF‐1α were purchased from Abcam (Cambridge, MA, USA). Antibodies for VEGF were obtained from Proteintech (Radnor, PA, USA).

### Samples collection and cell culture

Disc specimens were collected from twelve patients with disc perforation of human TMJ during partial discectomy. Control disc tissues were derived from seven patients with condylar fracture during joint arthroplasty. The protocol was approved by the Human Research Ethics Committee, School&Hospital of Stomatology, Wuhan University, and informed consents were also obtained from patients. Three perforated disc tissues and three control disc tissues were used for immunohistochemistry. The remaining disc samples were minced and digested with 0.25% of trypsin (HyClone, Logan, UT, USA) for 30 min. followed by type II collagenase for 2 hrs at 37°C. After washing, cells were grown in DMEM (HyClone) containing 10% of foetal bovine serum (FBS, HyClone), penicillin (100 units/ml, Hyclone) and streptomycin (100 μg/ml, Hyclone) in a humidified atmosphere containing 5% of CO_2_. Disc cells between the fourth and eighth passages were used for experiments.

HUVECs (ATCC, CRL, Rockefeller, MD, USA) were maintained in endothelial cell basal media‐2 (EGM‐2) Bullet kit media (Clonetics, BioWhittaker, San Diego, CA, USA) at 37°C in a humidified atmosphere containing 5% of CO_2_, and used for experiments at passages less than six.

### Generation of conditioned medium

Disc cells were seeded in a six‐well plates in DMEM containing 10% of foetal bovine serum. When cells were grown to 80–90% confluence, the medium was changed and cells were stimulated by HMGB1 in the absence or presence of signalling pathways inhibitors (anti‐VEGF antibody, BAY87‐2243, U0126, SB203580, SP600125) individually.

### Tube formation assay

Matrigel Matrix (BD Biosciences, Pittsburgh, PA, USA) was polymerized at 37°C for 30 min. in 24‐well plates. The HUVECs suspended in conditioned medium were seeded onto a layer of Matrigel Matrix. The tube formation was observed with photomicroscope(Olympus Optical C., Melviller, NY, USA), and each well was photographed**.**

### HUVECs migration assay

Migration activity was measured by transwell assay (Corning, Costar, Tewksbury, MA, USA). About 1 × 10^5^ HUVECs were added to upper chamber in 200 μl of 2% FBS DMEM complete medium. Lower chamber contained 200 μl conditioned medium. Plates were incubated for 24 hrs at 37°C in 5% of CO_2_. Cells were fixed in 3.7% of formaldehyde solution for 15 min. and stained with 0.05% of crystal violet in PBS for 15 min. Then, cells on upper side of filters were removed with cotton‐tipped swabs, and filters were washed with PBS. Cells on undersides of filters were examined and counted under a microscope.

### H&E and immunohistochemical staining

Disc specimens were fixed in 4% of paraformaldehyde for 24 hrs and then embedded in paraffin. Sagittal sections (4 μm) were cut with a LeicaRM2265 microtome (Leica, Wetzlar, Germany). Then, the sections were treated with haematoxylin and eosin (HE) staining. Immunohistochemical staining was performed with mouse anti‐human HIF‐1α. The sections were incubated with pepsin (DIG‐3009; Maixin, Fuzhou, China) for 30 min. at 37°C, and then incubated with 3% of H_2_O_2_ for 30 min. Non‐specific binding was blocked with a goat blocking serum. The sections were incubated with antibody at 4°C overnight in a humidified chamber. The sections were then washed with PBS and stained by antimouse streptavidin–peroxidase kit (SP‐9001, Zhongshan Golden Bridge Biotechnology Co., Ltd., Beijing, China). At last, the sections were coloured by reacting with 3,3‐diaminobenzidine (DAB‐0031, Maixin). Haematoxylin was used for counter‐staining for light microscopy.

### Cellular immunofluorescent staining

Perforated disc cells were fixed in 4% of paraformaldehyde for 30 min. Primary antibodies against HIF‐1α and VEGF were incubated in blocking buffer for overnight at 4°C. The cells were incubated with the corresponding secondary antibodies for 1 hr at 37°C, and the nuclei were stained with 40, 6‐diamidino‐2‐ phenylindole (DAPI) for 3 min. Cells were observed and photographed with a fluorescence microscope (Leica).

### RNA isolation and quantitative real‐time PCR

Total RNA from perforated disc cells was isolated using the Trizol (Takara, Otsu, Japan) and was reversely transcribed to cDNA according to the manufacturer's protocol (Takara). The qRT‐PCR reactions were performed on an ABI Prism 7500 Real‐time PCR System (Applied Biosystems, Foster City, CA, USA) with PrimeScript RT reagent kit using gDNA Eraser (TaKaRa), as described previously 18. Primer sequences were as follows: HIF‐1α (forward: AAATCTCCGTCCCTCAACCT, reverse: GAAAACTTGGCAACCTTGGA); VEGF (forward: ATCTGCATGGTGATGTTGGA, reverse: AAGGAGGAGGGCAGAATCAT); β‐actin (forward: TGGCACCCAGCACAATGAA, reverse: CTAAGTCATAGTCCGCCTAGAAGCA). Gene expression was calculated using the comparative cycle threshold method (2^−ΔΔCt^), and the housekeeping gene β‐actin was used as an internal reference.

### Western blot analysis

Proteins were separated by SDS‐PAGE electrophoresis, which was performed on 10% or 12% of polyacrylamide gels, and then electro‐transferred to a PVDF membrane (Millipore, Billerica, MA, USA). The membrane was blocked with 5% of non‐fat milk and then incubated with different primary antibodies, including HIF‐1α, VEGF, p‐Erk, total Erk, p‐p38, total P38, p‐JNK, total JNK, β‐actin, followed by horseradish–peroxidase‐labelled secondary antibody. Immunoreactive proteins were detected using chemiluminescence ECL system (Advansta, Menlo Park, CA, USA), as described previously 19.

### Statistical analysis

Analysis was conducted using Prism 5.0 (Graphpad software, San Diego, CA, USA). Comparisons between two independent groups were analysed by Mann–Whitney U‐test, as the variables were demonstrated non‐parametric distributions. Data were expressed as the mean ± S.E.M. *P* < 0.05 was considered statistically significant.

## Results

### HMGB1 promotes VEGF‐dependent angiogenesis in perforated disc cells of TMJ

**Figure 1:**
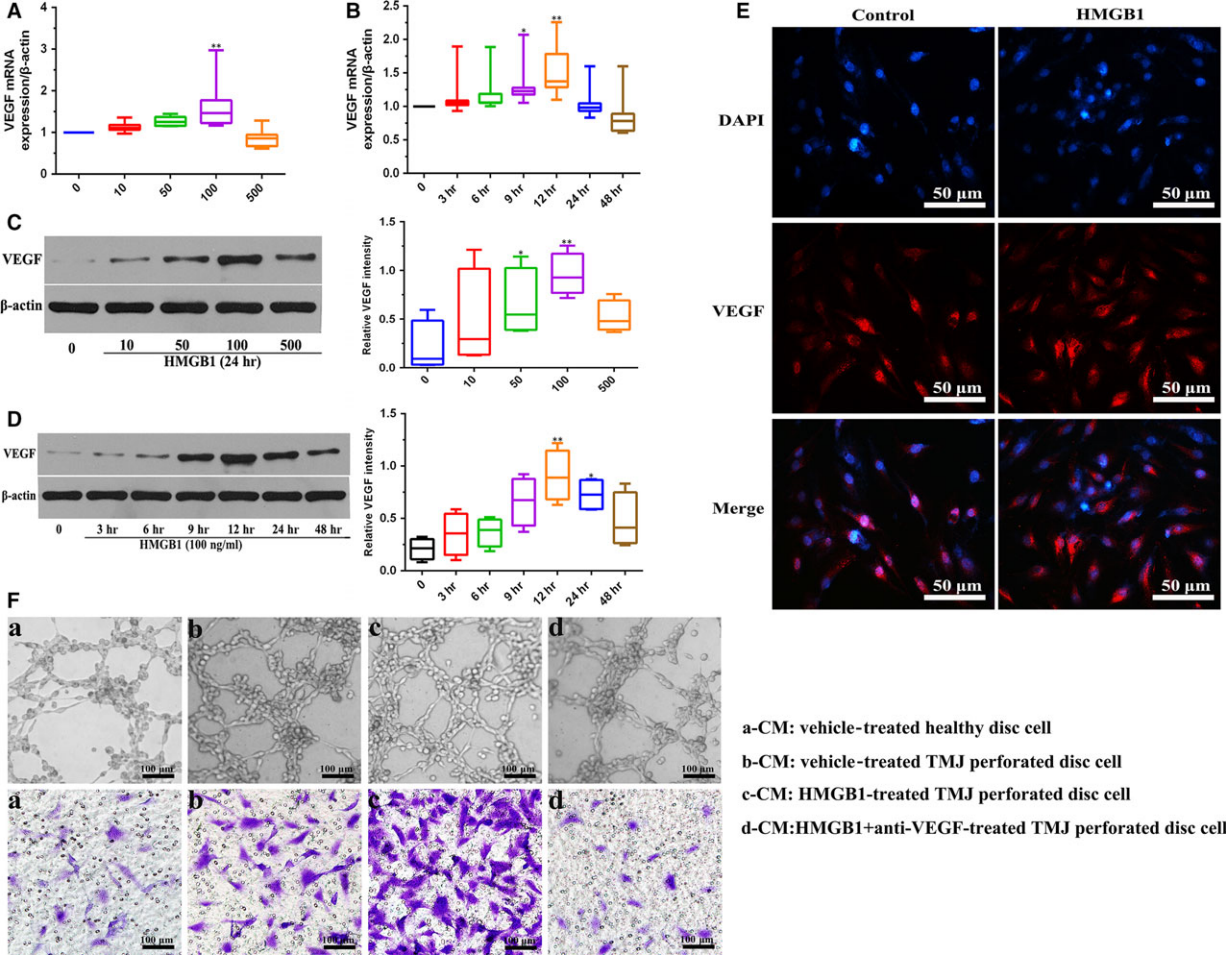
Effects of HMGB1 on VEGF expression at the mRNA and protein levels in perforated disc cells of human TMJ. Perforated disc cells were stimulated with HMGB1 for various concentrations (0–500 ng/ml), mRNA and protein expression levels of VEGF in these cells were detected by qRT‐PCR (**A**) and Western blot (**C**). β‐actin served as an internal control. (**B**,** D**) qRT‐PCR and Western blot analysis of VEGF in these cells after treatment with HMGB1 (100 ng/ml). β‐actin served as an internal control. (**E**) Immunofluorescent staining of VEGF in these cells with or without HMGB1 (100 ng/ml) treatment for 24 hrs. Scale bar = 50 μm. (**F**) Different conditioned medium (CM) (a, b, c, d) was collected for tube formation assay and migration of HUVECs. Scale bar = 100 μm. Box plots, 25th and 75th percentiles; horizontal solid lines, medians; horizontal bars, minimum and maximum. *P < 0.05 and **P < 0.01 compared to the group without HMGB1 stimulation.

VEGF is a potent pro‐angiogenic growth factor playing a pivotal role in angiogenesis. Hence, we investigated whether the expression of VEGF in perforated disc cells was raised after incubation of HMGB1. As shown in Figure 1A–D, When cells were incubated with HMGB1, the expression of VEGF was significantly increased in a concentration‐ and time‐dependent manner . Consistently, immunofluorescence staining exhibited that VEGF was expressed in perinuclear zone of perforated disc cells treated by 100 ng/ml HMGB1 for 24 hrs (Fig. 1E). Additionally, we compared tube formation and migration of HUVECs after the incubation of conditioned medium derived from HMGB1 (100 ng/ml, for 24 hrs)‐stimulated perforated disc cells with that using conditioned medium from vehicle‐treated perforated disc cells. Upon exposure of HUVECs to conditioned medium from HMGB1 (100 ng/ml, for 24 hrs)‐stimulated perforated disc cells, the numbers of tube formation and migration of cells increased significantly, as compared with those using conditioned medium from vehicle‐treated perforated disc cells. Moreover, pre‐treatment with VEGF antibodies inhibited HMGB1‐induced tube formation and migration of HUVECs (Fig. 1F). Accordingly, these results indicated that VEGF is involved in HMGB1‐induced angiogenesis in perforated disc cells of TMJ.

### Characterization of HMGB1‐induced expression and activation of HIF‐1α in perforated disc cells of TMJ

**Figure 2:**
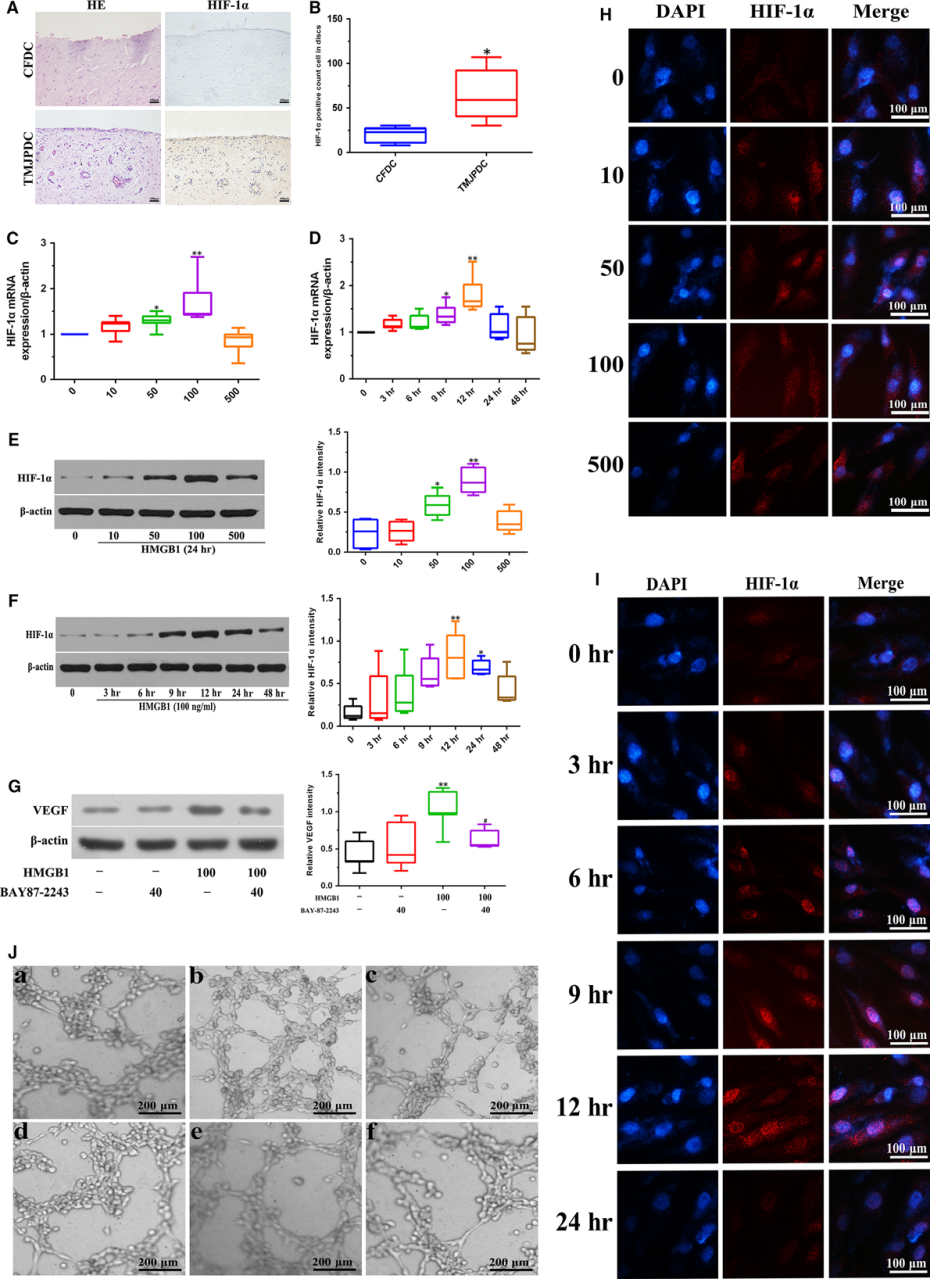
Effects of HMGB1 on expression and activation of HIF‐1α in perforated disc cells of human TMJ. (**A**,** B**) Disc tissues from patients with disc perforation and from patients with condylar fracture were compared using HE, immunohistochemical staining against HIF‐1α, and the number of HIF‐1α‐positive staining cells was analysed. (**C**,** E**) Perforated disc cells were treated with different doses of HMGB1 (0–500 ng/ml) for HIF‐1α mRNA and protein analysis at 24 hrs. β‐actin was used as a loading control. (**D**,** F**) These cells were incubated for various times (0–48 hrs) with HMGB1 (100 ng/ml). HIF‐1α mRNA and protein levels were determined by RT‐PCR or Western blotting, respectively. β‐actin was used as a loading control. (**G**) Perforated disc cells were pre‐treated with BAY87‐2243 (40 μM) for 1 hr and then incubated HMGB1 (100 ng/ml) for 24 hrs. VEGF protein levels were determined by Western blotting. β‐actin was used as a loading control. (**H**,** I**) Effect of HMGB1 on activity of HIF‐1α in perforated disc cells using immunofluorescence. Scale bar = 50 μm. (**J**) Perforated disc cells were pre‐treated with BAY87‐2243 (40 μM), U0126 (60 μM), SB203580 (40 μM) or SP600125 (60 μM) for 30 min, and then incubated with HMGB1 (100 ng/ml) for 24 hrs. Culture medium was then collected for tube formation of HUVECs. Box plots, 25th and 75th percentiles; horizontal solid lines, medians; horizontal bars, minimum and maximum.*P < 0.05 and **P < 0.01 compared to the group without HMGB1 stimulation. ^#^ *P* < 0.05 compared to the group treated with HMGB1 alone. a. Conditioned medium (CM) from vehicle‐treated perforated disc cells; b. CM from HMGB1‐treated perforated disc cells; c. CM from HMGB1 + BAY87‐2243‐treated perforated disc cells; d. CM from HMGB1 + U0126‐treated perforated disc cells; e. CM from HMGB1 + SB203580‐treated perforated disc cells; f. CM from HMGB1 + SP600125‐treated perforated disc cells.

As shown in Figure 2A, strong HIF‐1α expression was distributed in perforated discs of TMJ. When perforated disc cells were stimulated with various concentrations of HMGB1 (0–500 ng/ml) at 24 hrs, the expression levels of HIF‐1α mRNA and protein were subsequently increased in a dose‐dependent manner (Fig. 2C and E). Similarly, a time‐dependent increase of HIF‐1α expression occurred in these cells with the treatment of HMGB1 (100 ng/ml) (Fig. 2D and F). Besides, HIF‐1α was gradually translocated to the nuclear region of perforated disc cells when treated with HMGB1, indicating HMGB1 can mediate activation of HIF‐1α in these cells (Fig. 2H and I). Furthermore, application of BAY87‐2243 (HIF‐1α inhibitor) in these cells reduced HMGB1‐induced VEGF expression and tube formation of HUVECs (Fig. 2G and J). These results suggest that HMGB1 mediates activation of HIF‐1α in perforated disc cells of TMJ, and then initiates VEGF expression, thereby contributing to angiogenesis.

### HMGB1‐induced activation of HIF‐1α in perforated **disc cells of human TMJ** *via* Erk and JNK pathway

**Figure 3:**
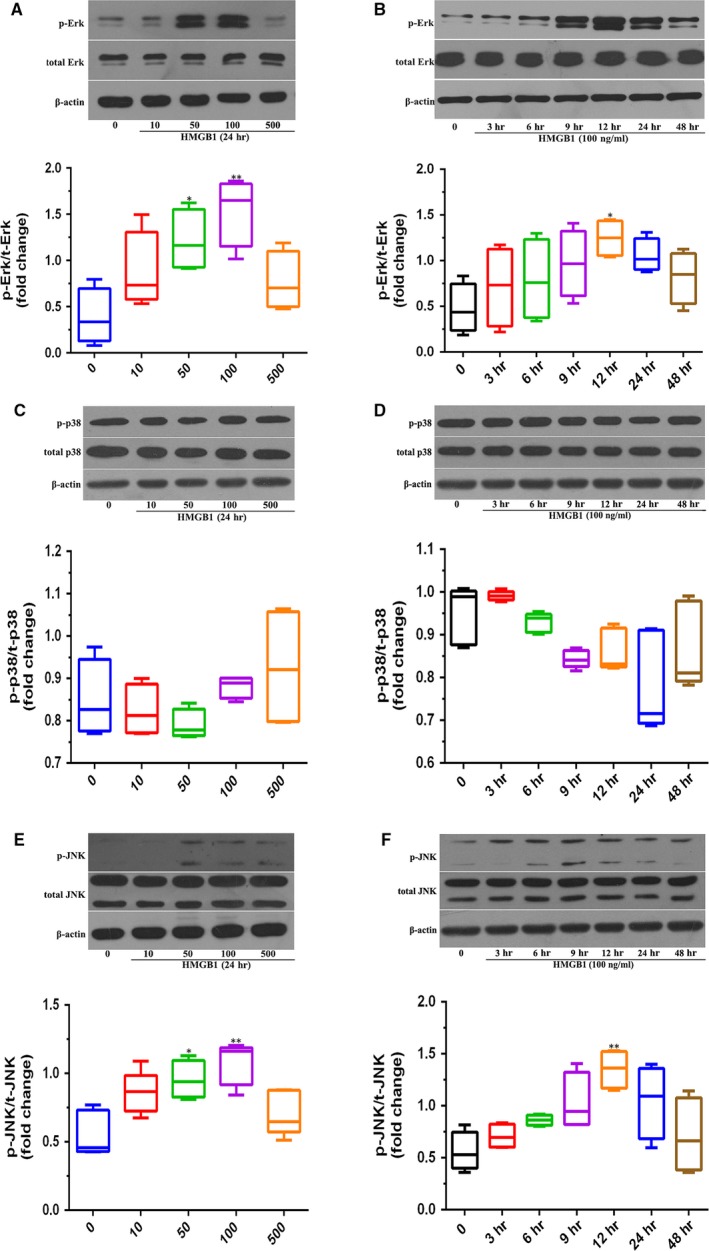
HMGB1‐activated Erk and JNK signalling pathways in perforated disc cells of human TMJ. (**A**,** C**,** E**) Perforated disc cells treated with different doses of HMGB1 for 24 hrs and expression of different signal proteins was detected by Western blot. (**B**,** D**,** F**) These cells were incubated for various times (0–48 hrs) with HMGB1 (100 ng/ml), and different signal protein levels were determined by Western blotting. β‐actin was used as the loading control. Box plots, 25th and 75th percentiles; horizontal solid lines, medians; horizontal bars, minimum and maximum. *P < 0.05 and **P < 0.01 compared to the group without HMGB1 stimulation.

To investigate the potential signalling pathway for HMGB1 to regulate angiogenesis in perforated disc cells of TMJ, we assessed the protein levels of Erk, JNK and P38 in these cells after the treatment of HMGB1. We stimulated perforated disc cells with HMGB1 at different concentrations (0–500 ng/ml) for 24 hrs or different times (0–48 hrs) at 100 ng/ml and detected the protein levels of Erk, JNK and P38 and their respective active forms (p‐Erk, p‐JNK and p‐P38) by Western blot. We found that HMGB1‐induced Erk and JNK phosphorylation in these cells in a dose‐ and time‐ dependent manner (Fig. 3A, B, E, F). However, no change of Erk, JNK, P38 and p‐P38 was detected (Fig. 3A–F).

**Figure 4:**
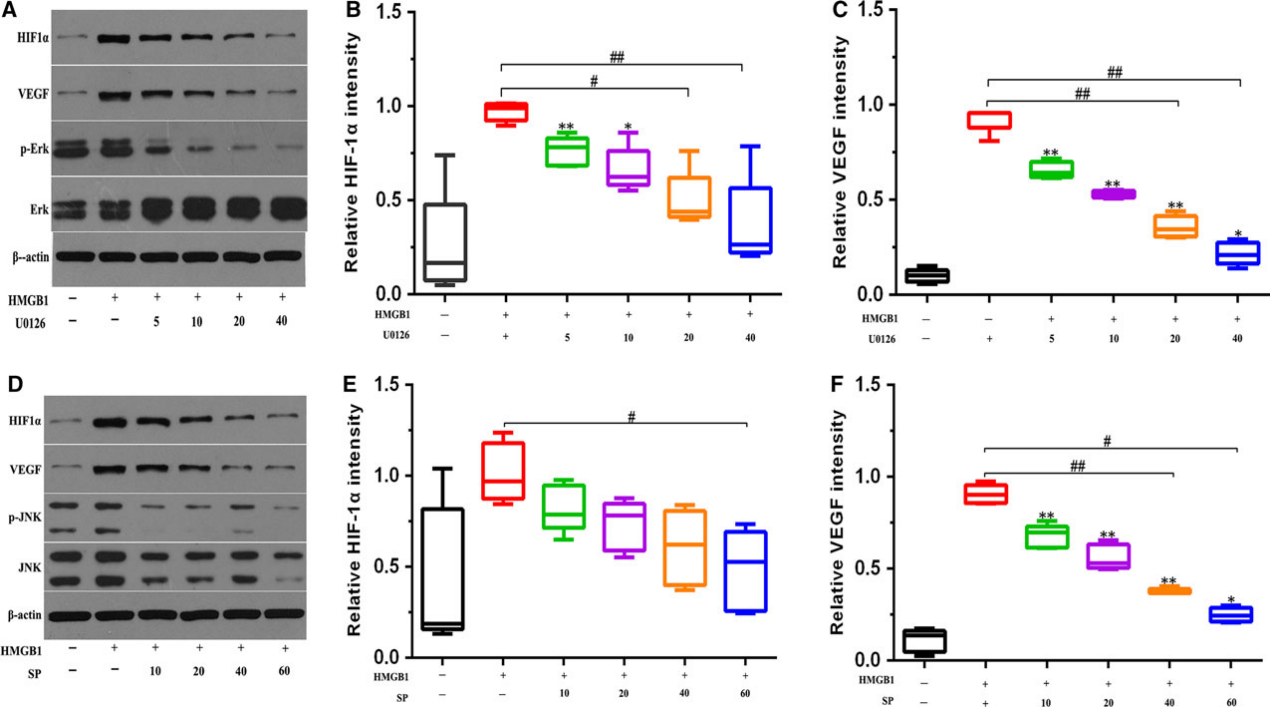
Inhibition of the Erk and JNK signalling pathway decreased HMGB1‐induced HIF‐1α and VEGF expression in perforated disc cells of human TMJ. Perforated disc cells were treated with Erk inhibitor (U0126) and JNK inhibitor (SP600125) for 30 min., followed by incubation of HMGB1 (100 ng/ml) for 24 hrs. (**A**,** D**) HIF‐1α and VEGF expression was assessed by Western blot analysis. β‐actin was used as the loading control. (**B**,** C**,** E**,** F**) Quantification of protein expression was performed using Images J software. Box plots, 25th and 75th percentiles; horizontal solid lines, medians; horizontal bars, minimum and maximum. *P < 0.05 and **P < 0.01compared to the group without HMGB1 stimulation. ^#^ *P* < 0.05 and ^##^ *P* < 0.01 compared to the group treated with HMGB1 alone.

**Figure 5:**
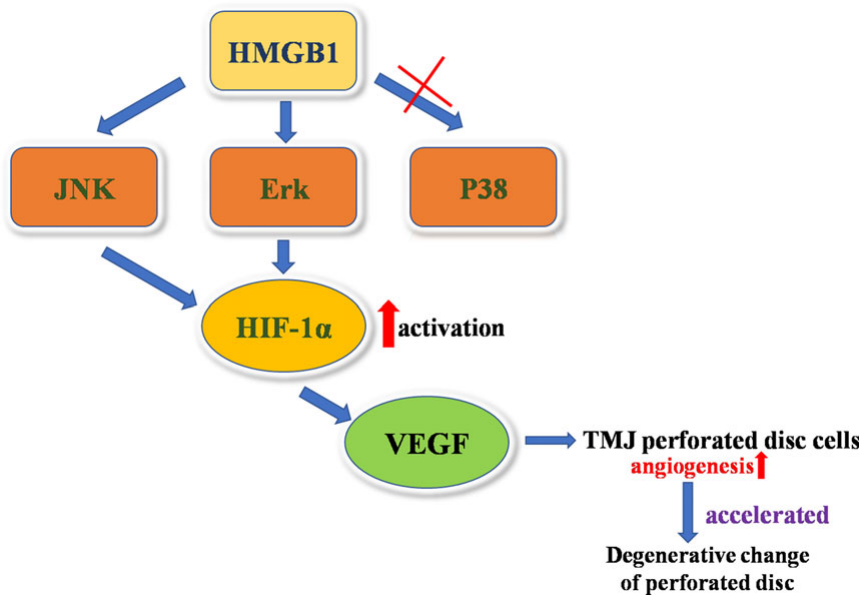
Schematic diagram of signalling pathways involved in HMGB1‐induced HIF‐1α expression and angiogenesis in human TMJ perforated disc cells. HMGB1 activates ERK, JNK pathway, leading to the up‐regulation of HIF‐1α, contributing to the VEGF expression and activity, promoting angiogenesis in human TMJ perforated disc cells.

Furthermore, as strong Erk and JNK activation was observed in response to HMGB1 stimulation, we assessed whether the use of specific Erk and JNK inhibitors could prevent HMGB1‐induced HIF‐1α expression and angiogenic effects in perforated disc cells. After pre‐treatment with JNK (SP600125), and Erk inhibitors (U0126) for 30 min., HMGB1‐induced HIF‐1α and VEGF productions in these cells were reduced in a dose‐dependent way (Fig. 4A and B). Results portend HMGB1 acting *via* Erk and JNK signalling pathways to promote HIF‐1α‐dependent angiogenesis in perforated disc cells. Moreover, the tube formation in HUVECs stimulated by HMGB1‐treated conditioned medium was significantly abolished when pre‐treating with U0126, SB203580, SP600125 (Fig. 2I). These results indicate that HMGB1 contributes to angiogenesis by up‐regulation of HIF‐1α and VEGF in perforated disc cells of TMJ *via* activation of Erk and JNK (Fig. 5).

## Discussion

TMJ disc, a fibro‐cartilaginous tissue, aids in joint motions by lubricating the surfaces of motion, decreasing incongruence between the articulating surfaces, and dissipating loads and impact within the joint 20. A variety of studies have shown that angiogenesis of pathological discs correlates with the severity of joint degeneration 21, 22, 23. Our previous study demonstrated the abundance of HMGB1 surrounding the perforated area of discs. HMGB1 has been involved in many angiogenesis‐related conditions, including cancer, wound healing, cornea neovascularization and ischaemia‐induced angiogenesis 23, 24. Accordingly, in this study, we tempted to explore the possible mechanisms underlying HMGB1 and deformed disc angiogenesis. We found that conditioned medium from HMGB1‐treated perforated disc cells aggravated the tube formation and migration of HUVECs. Moreover, our results showed that HIF‐1α was highly expressed in the perforated discs tissue and HMGB1 induced the elevation of HIF‐1α and VEGF in human TMJ perforated disc cells by activating ERK and JNK pathways.

Angiogenesis can contribute to synovitis, osteochondral damage, osteophyte formation and discs degeneration in patients with TMJ 25. A variety of molecules were described involved in the process of angiogenesis. VEGF, a potent vascular permeability factor, is pivotal in TMJ pathogenesis 26, 27 **.** Our previous study demonstrated high expression of VEGF in perforated disc tissue 28. We also found high expression of HMGB1 in perforated discs vicinity. Concurrent expression of these two factors impelled us to assume a potential correlation in them. Indeed, our *in vivo* study demonstrated that HMGB1 induced a time‐ and concentration‐dependent elevation of VEGF in perforated disc cells. Consistent with this observation, recent study showed that HMGB1 induces vascularization after peripheral ischaemia in diabetic mice through a VEGF‐dependent mechanism 29. In this study, we also observed that conditioned medium from HMGB1‐treated perforated disc cells mediated the tube formation and migration of HUVECs, while this phenomenon attenuated with the addition of VEGF antibodies, indicating HMGB1 induces VEGF‐dependent angiogenesis in perforated disc cells of human TMJ.

It has been established that the pathologic TMJ disc frequently accompanies with hypoxic environment and the cellular reaction to hypoxia is mainly driven by HIF‐1α 30. HIF‐1α can mediate VEGF gene expression by binding to its specific promoter region 31. Therefore, it is interesting to explore whether HIF‐1α regulates HMGB1‐induced VEGF expression in perforated disc cells. Our results showed that HIF‐1α was highly expressed in perforated discs tissue. To our knowledge, this is the first report demonstrating expression of HIF‐1α in TMJ discs. Furthermore, exogenous HMGB1 up‐regulated HIF‐1α mRNA and protein levels in these cells significantly. Besides, HMGB1 increased accumulation of HIF‐1α into nucleus, suggesting HMGB1 initiates activation of HIF‐1α in the perforated disc cells. Next, our findings showed that pre‐treatment with HIF‐1α inhibitor diminished HMGB1‐induced VEGF expression of perforated disc cells, tube formation of HUVECs, indicating HMGB1 mediates angiogenesis of perforated discs through up‐regulation of HIF‐1α‐dependent VEGF expression. Consistently, other studies performed by Park *et al*. reported a similar pattern of HMGB1‐HIF‐1α‐VEGF in the synovial fibroblasts from inflamed joint 15.

Erk, JNK and P38 as potential candidate signalling molecules have shown capacity for modulating the pathway of HMGB1‐HIF1α‐VEGF in the various studies 32, 33, 34. In this study, our results showed that blocking the Erk or JNK pathway with the specific inhibitor individually effectively reduces HMGB1‐induced HIF‐1α and VEGF production in the perforated disc cells, thereby abolishing tube formation and migration of HUVECs. Notably, P38 was not activated in these cells with the incubation of HMGB1 at the range of 10–500 ng/ml. One of the reasons for these discrepancies might be attributed from the divergent concentration of HMGB1 applied. Zabini *et al*. reported that a robust activation of P38 was only observed at 1 ng/ml of HMGB1 in pulmonary arterial endothelial cells 35. The other reasonable explanation is due to the unique characteristics of perforated disc cells from human TMJ.

Taken together, abundance of HMGB1 in perforated discs vicinity mediates activation of HIF‐1α in disc cells *via* Erk and JNK pathway and then, initiates VEGF secretion, thereby leading to disc angiogenesis and accelerating degenerative change of the perforated disc. These findings are helpful to illuminate the molecular mechanism of vascularization during the pathological degeneration of TMJ discs and disclose that HMGB1 is a potential therapeutic target for prevention of disc angiogenesis in TMJ.

## Conflict of interest

The authors confirm that there are no conflicts of interest.

## References

[1] Cholitgul W , Petersson A , Rohlin M , *et al* Clinical and radiological findings in temporomandibular joints with disc perforation. Int J Oral Maxillofac Surg. 1990; 19: 220–5. 2120363 10.1016/s0901-5027(05)80396-0

[2] Widmalm SE , Westesson PL , Kim IK , *et al* Temporomandibular joint pathosis related to sex, age, and dentition in autopsy material. Oral Surg Oral Med Oral Pathol. 1994; 78: 416–25. 7800370 10.1016/0030-4220(94)90031-0

[3] Muñoz‐Guerra MF , Rodríguez‐Campo FJ , Hernández VE , *et al* Temporomandibular joint disc perforation: long‐term results after operative arthroscopy. J Oral Maxillofac Surg. 2013; 71: 667–76. 23507320 10.1016/j.joms.2012.12.013

[4] Rees LA . The structure and function of the mandibular joint. Br Dent J. 1954; 96: 125–33.

[5] Mazzonetto R , Spagnoli DB . Long‐term evaluation of arthroscopic discectomy of the temporomandibular joint using the Holmium YAG laser. J Oral Maxillofac Surg. 2001; 59: 1018–23. 11526570 10.1053/joms.2001.25829

[6] Loreto C , Almeida LE , Migliore MR , *et al* TRAIL, DR5 and caspase 3‐dependent apoptosis in vessels of diseased human temporomandibular joint disc. An immunohistochemical study. Eur J Histochem. 2010; 54: 175–179. 10.4081/ejh.2010.e40 PMC3167309 20839416

[7] Koch AE , Harlow LA , Haines GK , *et al* Vascular endothelial growth factor. A cytokine modulating endothelial function in rheumatoid arthritis. J Immunol. 1994; 152: 4149–56. 7511670

[8] Agrawal A , Guttapalli A , Narayan S , *et al* Normoxic stabilization of HIF‐1alpha drives glycolytic metabolism and regulates aggrecan gene expression in nucleus pulposus cells of the rat intervertebral disk. Am J Physiol Cell Physiol. 2007; 293: C621–31. 17442734 10.1152/ajpcell.00538.2006

[9] Wang Y , Zhong J , Zhang X , *et al* The Role of HMGB1 in the pathogenesis of type 2 diabetes. J Diabetes Res. 2016; 2016: 1–11. 10.1155/2016/2543268 PMC5215175 28101517

[10] Kang R , Chen R , Zhang Q , *et al* HMGB1 in health and disease. Mol Aspects Med. 2014; 40: 1–116. 25010388 10.1016/j.mam.2014.05.001 PMC4254084

[11] Tang D , Kang R , Livesey KM , *et al* High‐mobility group box 1 is essential for mitochondrial quality control. Cell Metab. 2011; 13: 701–11. 21641551 10.1016/j.cmet.2011.04.008 PMC3293110

[12] Andersson U , Tracey KJ . HMGB1 is a therapeutic target for sterile inflammation and infection. Annu Rev Immunol. 2011; 29: 139–62. 21219181 10.1146/annurev-immunol-030409-101323 PMC4536551

[13] Seidu RA , Wu M , Su Z , *et al* Paradoxical role of high mobility group box 1 in glioma: a suppressor or a promoter? Oncol Rev. 2017; 11: 325. 28382190 10.4081/oncol.2017.325 PMC5364998

[14] Mitola S , Belleri M , Urbinati C , *et al* Cutting edge: extracellular high mobility group box‐1 protein is a proangiogenic cytokine. J Immunol. 2006; 176: 12–5. 16365390 10.4049/jimmunol.176.1.12

[15] Park SY , Lee SW , Kim HY , *et al* HMGB1 induces angiogenesis in rheumatoid arthritis *via* HIF‐1alpha activation. Eur J Immunol. 2015; 45: 1216–27. 25545169 10.1002/eji.201444908

[16] Biscetti F , Flex A , Pecorini G , *et al* The role of high‐mobility group box protein 1 in collagen antibody‐induced arthritis is dependent on vascular endothelial growth factor. Clin Exp Immunol. 2016; 184: 62–72. 26671547 10.1111/cei.12758 PMC4778102

[17] Feng Y , Fang W , Li C , *et al* The expression of high‐mobility group box protein‐1 in temporomandibular joint osteoarthritis with disc perforation. J Oral Pathol Med. 2016; 45: 148–52. 26082301 10.1111/jop.12336

[18] Ke J , Long X , Liu Y , *et al* Role of NF‐kappaB in TNF‐alpha‐induced COX‐2 expression in synovial fibroblasts from human TMJ. J Dent Res. 2007; 86: 363–7. 17384033 10.1177/154405910708600412

[19] Xu J , Liu Y , Deng M , *et al* MicroRNA221‐3p modulates Ets‐1 expression in synovial fibroblasts from patients with osteoarthritis of temporomandibular joint. Osteoarthritis Cartilage. 2016; 24: 2003–11. 27349463 10.1016/j.joca.2016.06.011

[20] Jonsson G , Eckerdal O , Isberg A . Thickness of the articular soft tissue of the temporal component in temporomandibular joints with and without disk displacement. Oral Surg Oral Med Oral Pathol. 1999; 87: 20–6. 10.1016/s1079-2104(99)70289-1 9927075

[21] Kepler CK , Ponnappan RK , Tannoury CA , *et al* The molecular basis of intervertebral disc degeneration. Spine J. 2013; 13: 318–30. 23537454 10.1016/j.spinee.2012.12.003

[22] Miller D , DeSutter C , Scott A , *et al* Vascular structure and function in the medial collateral ligament of anterior cruciate ligament transected rabbit knees. J Orthop Res. 2014; 32: 1104–10. 24909758 10.1002/jor.22643

[23] Kim JS , Ali MH , Wydra F , *et al* Characterization of degenerative human facet joints and facet joint capsular tissues. Osteoarthritis Cartilage. 2015; 23: 2242–51. 26117175 10.1016/j.joca.2015.06.009 PMC4663154

[24] He C , Sun Y , Ren X , *et al* Angiogenesis mediated by toll‐like receptor 4 in ischemic neural tissue. Arterioscler Thromb Vasc Biol. 2013; 33: 330–8. 23241411 10.1161/ATVBAHA.112.300679

[25] Sato J , Segami N , Yoshitake Y , *et al* Correlations of the expression of fibroblast growth factor‐2, vascular endothelial growth factor, and their receptors with angiogenesis in synovial tissues from patients with internal derangement of the temporomandibular joint. J Dent Res. 2003; 82: 13–18. 10.1177/154405910308200406 12651930

[26] Murata M , Yudoh K , Masuko K . The potential role of vascular endothelial growth factor (VEGF) in cartilage: how the angiogenic factor could be involved in the pathogenesis of osteoarthritis? Osteoarthritis Cartilage. 2008; 16: 279–86. 17945514 10.1016/j.joca.2007.09.003

[27] Leonardi R , Lo Muzio L , Bernasconi G , *et al* Expression of vascular endothelial growth factor in human dysfunctional temporomandibular joint discs. Arch Oral Biol. 2003; 48: 185–92. 12648555 10.1016/s0003-9969(02)00207-8

[28] Xu J , Cai H , Meng Q , *et al* IL‐1beta‐regulating angiogenic factors expression in perforated temporomandibular disk cells *via* NF‐kappaB pathway. J Oral Pathol Med. 2016; 45: 605–12. 26775638 10.1111/jop.12420

[29] Biscetti F , Straface G , De Cristofaro R , *et al* High‐mobility group box‐1 protein promotes angiogenesis after peripheral ischemia in diabetic mice through a VEGF‐dependent mechanism. Diabetes. 2010; 59: 1496–505. 20200317 10.2337/db09-1507 PMC2874711

[30] Mino‐Oka A , Izawa T , Shinohara T , *et al* Roles of hypoxia inducible factor‐1alpha in the temporomandibular joint. Arch Oral Biol. 2017; 73: 274–81. 27816790 10.1016/j.archoralbio.2016.10.028

[31] Bawa‐Khalfe T , Yang FM , Ritho J , *et al* SENP1 regulates PTEN stability to dictate prostate cancer development. Oncotarget. 2017; 8: 17651–64. 27852060 10.18632/oncotarget.13283 PMC5392276

[32] Kang R , Zhang Q , Hou W , *et al* Intracellular Hmgb1 Inhibits Inflammatory Nucleosome Release and Limits Acute Pancreatitis in Mice. Gastroenterology. 2014; 146: 1097–107. 24361123 10.1053/j.gastro.2013.12.015 PMC3965592

[33] Ohmori H , Luo Y , Kuniyasu H . Non‐histone nuclear factor HMGB1 as a therapeutic target in colorectal cancer. Expert Opin Ther Targets. 2011; 15: 183–93. 21204727 10.1517/14728222.2011.546785

[34] Hajishengallis G , Lambris JD . Crosstalk pathways between Toll‐like receptors and the complement system. Trends Immunol. 2010; 31: 154–63. 20153254 10.1016/j.it.2010.01.002 PMC2849859

[35] Zabini D , Crnkovic S , Xu H , *et al* High‐mobility group box‐1 induces vascular remodelling processes *via* c‐Jun activation. J Cell Mol Med. 2015; 19: 1151–61. 25726846 10.1111/jcmm.12519 PMC4420616

